# Chronic Diarrhea Due to *Aeromonas hydrophila* in an Immunosuppressed Patient with a Pancreas–Kidney Transplant

**DOI:** 10.3390/pathogens12091151

**Published:** 2023-09-11

**Authors:** Pablo Solís-Sánchez, Marta Fernández-Martínez, Emilio Rodrigo-Calabia, Carlos Ruiz de Alegría-Puig

**Affiliations:** 1 Internal Medicine Service, University Hospital Marqués de Valdecilla-IDIVAL, 39008 Santander, Spain; 2 Microbiology Service, University Hospital Marqués de Valdecilla-IDIVAL, 39008 Santander, Spain; 3 Nephrology Service, University Hospital Marqués de Valdecilla-IDIVAL, 39008 Santander, Spain; 4 Microbiology Service, University Hospital Marqués de Valdecilla-IDIVAL-CIBERINFEC, 39008 Santander, Spain; carlos.ruizdealegria@scsalud.es

**Keywords:** *Aeromonas*, *A. hydrophila*, pancreas–kidney transplant, chronic diarrhea

## Abstract

The genus *Aeromonas* belongs to the *Aeromonadaceae* family. A patient with a pancreas–kidney transplant had multiple episodes of abdominal sepsis after surgery. *Aeromonas hydrophila* was isolated in the ascitic and biliary fluid drains. After discharge, the patient had several diarrhea episodes, and *A. hydrophila* was isolated in four stool samples. We decided to test whether the one strain that we initially isolated in ascitic fluid was the same that appeared in the successive stool samples. Five isolates of *A. hydrophila* were found in the patient. Identification was performed using the MALDI-TOF system and confirmed via multiplex PCR. The analysis of the REP-PCR fingerprint patterns showed one cluster and confirmed that all isolates were related. We also demonstrated the virulent character of this species associated with genes encoding different toxins (act, alt, ast, hlyA, and aerA). The virulence of this species is associated with the expression of genes that encode different toxins, structural proteins, and metal-associated proteins. This case report highlights the severity of this disease, especially in immunocompromised patients, and its adequate treatment.

## 1. Introduction

The *Aeromonas* genus belongs to the *Aeromonadaceae* family, a group of Gram-negative, oxidase-positive, and catalase-positive bacteria [1]. The first time *Aeromonas* spp. was considered a human pathogen was in 1954, when it was isolated from the blood, lungs, liver, spleen, urine, cerebrospinal fluid, and necrotic parts of some striated muscles of an immunocompromised woman [2]. Over the past years, *Aeromonas* spp. has received increasing attention as an emergent agent of foodborne illness. It inhabits a variety of niches including aquatic habitats, aquatic animals, soils, terrestrial animals, and human beings. Most of these pathogens come into human systems through ingestion of water or food contaminated with *Aeromonas* spp. For example, in India, *Aeromonas* spp. has been detected in 13.4% of animal-origin food samples [3]. These bacteria grow well at higher temperatures; so, an increase in bacterial load may be attributed to a rise in temperature in freshwater environments [4].

*Aeromonas* spp. infections are mostly polymicrobial, and there can be competition and cooperation between bacterial cells [4]. They cause a wide variety of diseases in humans, especially gastroenteritis, septicemia, and wound infections [5]. The diarrhea caused varies from a mild form to a Shigella-like dysentery, or a severe, watery, cholera-like diarrhea [6]. It is also implicated in other extraintestinal pathologies, usually biliary disease, in both immunocompetent and immunosuppressed patients [7]. It can be a cause of sepsis, especially in patients with underlying hepatobiliary and malignant disease. In our healthcare district, the most prevalent species was *Aeromonas caviae* (78.7%) [8]. *Aeromonas hydrophila* and *Aeromonas veronii* are the most frequent in the biliary system. 

We present a case report of chronic diarrhea due to *A. hydrophila* and discuss its importance in gastrointestinal tract infections. It has recently been a subject of debate due to alarming publications on the increase in the virulence gene profiles [9]. In our study, we used these genes to identify one strain found in different samples from a single patient.

## 2. Case Presentation

A 42-year-old female with a 30-year history of type 1 diabetes mellitus developed several micro- and macroangiopathic complications, including chronic kidney disease. After initiation of renal replacement therapy with twice-weekly hemodialysis, she required combined pancreas and kidney transplantation. There were multiple complications. Renal artery thrombosis influenced graft failure and transplantectomy. A pancreatic fistula formed, which led to abdominal sepsis. A drain was placed near the intra-abdominal fistula, and several microorganisms were isolated: *Escherichia coli*, *Clostridium perfringens*, and *Enterococcus faecium*. Multiple courses of antibiotics were prescribed to treat these infections, including meropenem, metronidazole, and vancomycin. Afterwards, *A. hydrophila* was isolated in the ascitic and biliary fluid drainage; it was decided not to treat it because she was asymptomatic. 

Over the following months, the patient experienced intermittent episodes of watery diarrhea with no fever, blood, or mucous. Laboratory data were significant for a serum albumin level of 3.1 g/dL. Other evaluations including anti-transglutaminase IgA and anti-endomysial IgA were unremarkable. A colonoscopy showed normal colon mucosa. A stool examination revealed no parasites and negative occult blood. *A. hydrophila* was isolated again in four stool samples taken on an outpatient basis. In accordance with the antibiotic susceptibility test, trimethoprim–sulfamethoxazole (MIC < 2) was used to treat the infection, but she had a poor functional recovery. Since then, the patient has had several consultations to study this chronic diarrhea, with no new bacterial or viral isolations.

**Figure 1:**
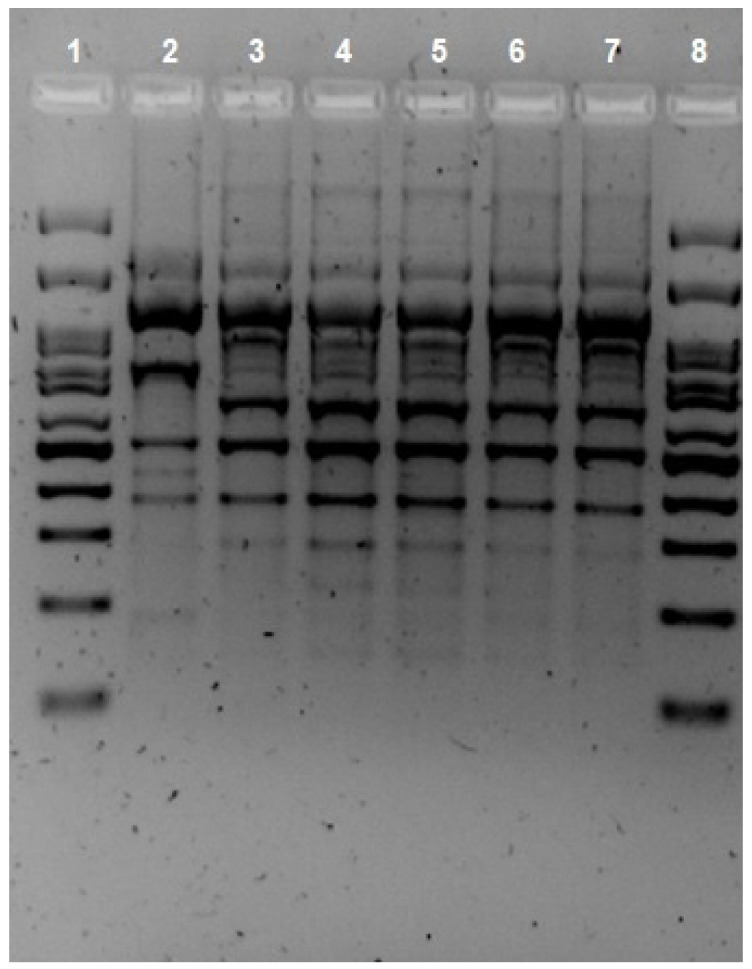
REP-PCR fingerprint patterns of the *Aeromonas hydrophila* isolates. Lines 1 and 8 Ladder, Lines 2 *Aeromonas hydrophila*. Line 3 sample from peritoneal drainage. Lines 4, 5, 6, and 7 samples from feces.

Given that there were isolations of *A. hydrophila* in different locations in the gastrointestinal tract, it was decided to test whether the strain initially isolated in the ascitic fluid was the same as that found in successive stool samples. Indeed, five isolates of *A. hydrophila* were found in the patient over a seven-month period. The first one was isolated from peritoneal drainage and the following four from stool. The protocol for isolates from feces at the Marqués de Valdecilla University Hospital clinical microbiology laboratory entails culture in BD Yersinia Selective Agar (CIN Agar; BD, Heidelberg, Germany) and incubation at 37 °C for 24 h. The peritoneal drainage was cultured in chocolate agar (Oxoid, Altrincham, UK) and MacConkey agar (Oxoid, Altrincham, UK) and incubated at 37 °C for 24 h, as per protocol. Bacteria susceptibility testing was performed with the Vitek2 system (bioMerieux, Craponne, France) using VITEK^®^ 2 AST cards (bioMerieux, Craponne, France). Identification was performed using the MALDI-TOF system (Vitek-MS^®^, BioMerieux, Craponne, France) and confirmed via multiplex PCR, developed by Persson et al. [10]. The clonal relationship of the isolates was evaluated using repetitive extragenic palindromic PCR (REP-PCR), as described by Vila et al., using the primers created by us [11]. Two isolates were clonally related when two or more different bands were observed on visual inspection. The analysis of the REP-PCR fingerprint patterns (Figure 1) showed one cluster and confirmed that all isolates were related. In addition, identical results on susceptibility tests supported this assertion, as all isolates showed sensitivity to ciprofloxacin (MIC < 0.25) and trimethoprim–sulfamethoxazole (MIC < 2) and resistance to β-lactams [12]. Five virulence-associated genes (act, alt, ast, hlyA, and aerA) were found via PCR using the primers described by Hoel et al. [13]. Although these genes do not directly imply that they can cause clinical symptoms of infection, there is some evidence of a correlation between these toxin genes and their virulence trait [14].

## 3. Discussion

This case report describes the isolation of the *A. hydrophila* strain in ascitic and biliary fluid drainage and then in four stool samples and highlights the severity of this disease, especially in immunocompromised patients with hepatobiliary disease. The pathogenesis of *Aeromonas*-mediated infections is multifactorial, and the role of the virulence determinants in human infections is associated with the expression of genes that encode different toxins, structural proteins, and metal-associated proteins. Genes encoding thermolabile and thermostable cytotonic (alt and ast), cytotoxic (act), and hemolytic enterotoxins (hylA and aerA) have been characterized. The role of the three enterotoxins (alt, ast, and act) in causing *A. hydrophila*-induced gastroenteritis in an animal model was established, with the greatest contribution from the cytotoxic enterotoxin act [15]. Furthermore, the type III secretion system (T3TSS or TTSS) may deliver a range of toxins into the host cell [16]. Gene transfers occur through conjugation and transformation, in which type IV pili play a vital role [17].

The expression of peritrichous flagella encoded by the fla gene cluster enhances eukaryotic cells’ adherence and invasiveness [16,18]. Polar flagella allow swimming motility in liquid medium, while lateral flagella offer swarming motility in a solid medium [19]. In fact, mutation in either flaA or flaB does not affect the development of flagellum but reduces the adherence and motility by approximately 50% [20]. These data also support the deduction that both flagellar types enhance the biofilm formation of *Aeromonas* spp. on surfaces. Bacterial flagella and pili play important roles in gastric pathogenicity. Lateral flagella have been reported to have a correlation with chronic dysentery [20,21].

Several investigators have identified cholera-like-cytotonic enterotoxins in *Aeromonas* spp. culture filtrates that could be responsible for fluid secretion in the small intestine of animals without causing degeneration of crypts and villi of the small intestine [22]. In the study by Lee et al., the alt-gene-encoding heat-labile cytotonic enterotoxin was highly prevalent, whereas the ast-gene-encoding heat-stable cytotonic enterotoxin was not detected in any of the isolates [15]. The study by Albert et al. indicated that *Aeromonas* spp. isolates positive with both alt and ast genes might synergistically cause severe diarrhea [23,24].

One of the most potent virulence factors is a 52-kDa cytotoxic enterotoxin encoded by the act gene [25]. Act can bind cholesterol, which occurs only if the hydroxyl group of cholesterol is unmodified [22]. This binding to cholesterol facilitates the aggregation of act in lipid rafts, where it might interact with a host cell receptor or become internalized via endocytosis [22]. It generates an inflammatory response in host cells and promotes the degeneration of villi and mucus-producing cells, which may be related to cases of bloody diarrhea in humans. Act is optimally expressed at 37 °C and at pH 7.0 and is thus produced in greater amounts in vivo than in the external environment [26]. It has been shown to upregulate the expression of genes encoding proinflammatory cytokines (TNF-α, IL-1, and IL-6) and inducible nitric oxide synthase (iNOS) in murine macrophages [22]. Act also can activate the arachidonic acid metabolism via induction of phospholipase A2 (PLA2) and cyclooxygenase-2 (COX-2), with subsequent activation of adenylate cyclase and production of cAMP [22]. These mediators could be responsible for act-associated gastroenteritis. In the presence of high amounts of iron, there is a ferric uptake regulatory (fur) gene that repressed act gene expression [27].

*Aeromonas* spp. has hemolytic activities due to the presence of hemolysin (hylA) and aerolysin (aerA) genes [28]. Despite the significant differences between act and aerA, the two toxins are cytotoxic and hemolytic in nature, and both form pores in eukaryotic cell membranes [22,25]. The pore-forming action of aerolysin is well characterized: it binds glycosylphosphatidylinositol anchors, which might facilitate aggregation of the toxin on the plasma membrane and subsequent pore formation [29]. *Aeromonas* spp. can produce different hemolysins [30]. The α-hemolysins produce reversible effects and incomplete lysis of erythrocytes, while the ß-hemolysins produce holes in cell membranes, causing complete destruction of erythrocytes by osmotic enlargement [31,32].

A recent study identified the plasmid-encoded expression of two Shiga toxin genes (stx1 and stx2) in *Aeromonas* genus [33,34]. These toxins produced from enterohemorrhagic *E. coli* strains represent a horizontal transfer mechanism [35]. They can cause diarrhea, hemorrhagic colitis, and hemolytic uremic syndrome [36]. *Aeromonas* spp. also produces proteases, which can cause tissue damage, overcome host cell defenses, and provide nutrients for bacterial cell proliferation [31,37,38]. The three major types of proteases are a heat-labile serine protease, a heat-stable EDTA-sensitive protease, and a heat-stable EDTA-insensitive protease [22,31]. In addition, some aminopeptidases might function specifically to activate act and/or aerA [22]. Certain metalloproteases may interfere with host coagulation by cleaving prothrombin into its activated form, thrombin [39].

Its capacity for colonization in places such as the gallbladder has been linked to metallostasis, a biological process to obtain metals such as iron [40]. Metal ions are essential for the correct function of microbial biological processes; thus, the low concentration of free iron is an evolved host defense [41]. To obtain iron, these species synthesize and excrete Fe^+3^ specific ligands of low molecular mass, collectively known as siderophobes. Most species of *Aeromonas* genus produce the siderophobe, amonabactin [22,31]. The bacterial metal homeostasis is also related to metallochaperones, proteins that add metal ions to specific enzymes. One of the most studied metallochaperones is the nickel-binding protein HypA, previously described in the human pathogens *Escherichia coli* and *Helicobacter pylori* [42]. HypA participates in nickel-dependent hydrogenases’ and ureases’ maturation, and it could be associated with acid tolerance [42]. Resistance to acidic environments can be a great advantage for pathogens because reactive oxygen species (ROS) production is a defense mechanism against pathogens after phagocytosis by macrophages [4]. HypA genes are widely conserved in certain species like *A. hydrophila* and *A. veronii,* among others [40].

Other virulence factors include lipases [21,31], adhesins [43], nucleases [44], pore forming toxins [45], and catalysts [4].

By far, the most common disease associated with *Aeromonas* spp. infection is gastroenteritis, which varies from a mild self-limiting watery diarrhea to a more severe invasive Shigella-like dysenteric form. Several epidemiological studies have connected *Aeromonas* spp. to traveler’s diarrhea. Chronic diarrhea, caused by *A. hydrophila* or *A. caviae* and exceeding one year in duration, has also been reported [46]. Hematologic cancer patients and patients with gastrointestinal tumors are more likely to be infected by *Aeromonas* spp. Any portion of the colon may be affected, mostly the ascending or transverse sections; therefore, in certain cases, *Aeromonas*-segmental-colitis may seem similar to ischemic colitis or Crohn’s disease [47]. It can also cause intramural intestinal hemorrhage including small bowel obstruction [48].

The second most common *Aeromonas*-related disease is skin and soft tissue infection, which can range from mild problems like pustular lesions to dangerous conditions that can cause morbidity in an infected person, such as cellulitis, necrotizing fasciitis, myonecrosis, septic arthritis, and septic shock [49]. Another common manifestation is *Aeromonas*-associated wound infections [50]. There can be a transfer of bacteria from the gastrointestinal tract to the blood circulatory system. Sepsis is more prevalent in immunocompromised conditions, especially those with hematological malignancy, and *Aeromonas*-contaminated catheters may serve as a point of entry into human blood [51].

Most cases of *Aeromonas*-associated diarrhea are self-limited and can be managed with supportive therapy, including oral and intravenous rehydration [35]. Antibiotics may be used to treat severe diarrhea or bacteriemia. It is also indicated in patients with a history of immunosuppression. *Aeromonas* spp. is usually uniformly resistant to penicillin due to inducible chromosomal β-lactamases. However, they are susceptible to aminoglycosides, sulfa drugs, second–fourth generation cephalosporins, carbapenems, fluoroquinolones, and tetracyclines [52,53]. Three major classes of β-lactamases are present in *Aeromonas* spp.: C cephalosporinase, D penicillinase, and a class B metallo-β-lactamase (MBL) [54]. Among these, MBL, which works against carbapenems, are of major concern. CphA-encoded metallo-β-lactamase possesses an unusual spectrum of activity because it hydrolyzes carbapenems but not penicillins or cephalosporins [55]. Plasmids serve as a platform on which useful resistance genes are assembled and subsequently disseminated [56]. These infections are treatable with monotherapy, and studies with combination therapy do not show better outcomes [57]. Empiric therapy with a fluoroquinolone, third-generation cephalosporin, and/or TMP-SMX would provide reasonable antimicrobial coverage. Fluroquinolones should be considered as the first-choice therapy. They have been shown to be active with samples of *A. hydrophila*, *A. caviae,* and *A. veronii*, both in in vitro studies and in vivo models, having MICs less than 1 mg/mL in 90% of the samples evaluated [5]. However, fluoroquinolones should not be used in treating pediatric patients [58]. For severe soft-tissue infection, surgical debridement is recommended with adequate antimicrobial chemotherapy, and hyperbaric oxygen therapy may be effective [55]. High fatality rates were seen in patients with bacteremia, sepsis, severe soft-tissue infection, or pneumonia [55]. On the other hand, patients with diarrhea and cholangitis usually were associated with a good outcome if rational antimicrobial agents were administered [55].

## 4. Conclusions

In our case report, we hypothesize that *A. hydrophila* colonized the common bile duct and then it reached the intestines through fistulas and surgeries that caused intestinal motility disorders. We must consider this pathogen as a possible cause of chronic disease. Moreover, we postulate that the REP-PCR-based DNA fingerprint technique may be a rapid typing method for use in epidemiological studies of isolates belonging to the *Aeromonas* genus.

We also demonstrated the hypothetical virulent character of this species. According to Wang et al., screening for specific enterotoxin genes is suggested to characterize virulence factors [30]. Many virulence factors have been characterized from *Aeromonas* spp., especially from *A. hydrophila*, the main causative organism of diarrhea and sepsis in humans and in animals. This highlights the severity of this disease, especially in immunocompromised patients, and its adequate treatment. Strategies to promote rational antimicrobial treatment are necessary to reduce antibiotic resistance and its spread by plasmid-mediated horizontal gene transfer.

## Data Availability

No new data were created or analyzed in this study. Data sharing is not applicable to this article.

## References

[1] Janda J.M., Abbott S.L. (2010). The genus *Aeromonas*: Taxonomy, pathogenicity, and infection. Clin. Microbiol. Rev..

[2] Caselitz F.H. (1996). How the *Aeromonas* story started in medical microbiology. Med. Microbiol. Lett..

[3] Kumar A., Bachhil V.N., Bhilegaonakar K.N., Agarwal R.K. (2000). Occurrence of enterotoxigenic *Aeromonas* species in foods. J. Commun. Dis..

[4] Bhowmick U.D., Bhattacharjee S. (2018). Bacteriological, Clinical and Virulence Aspects of *Aeromonas*-associated Diseases in Humans. Pol. J. Microbiol..

[5] Parker J., Shaw J. (2011). *Aeromonas* spp. clinical microbiology and disease. J. Infect..

[6] Citterio B., Biavasco F. (2015). *Aeromonas hydrophila* virulence. Virulence.

[7] Chao C.M., Lai C.C., Tang H.J., Ko W.C., Hsueh P.-R. (2013). Biliary tract infections caused by *Aeromonas* species. Eur. J. Clin. Microbiol. Infect. Dis..

[8] Ruíz de Alegría-Puig C., Fernández-Martínez M., Pintos-Fonseca A. (2021). Epidemiology of *Aeromonas* spp. isolated from stool in a tertiary hospital in Cantabria, Northern Spain, in the last five years. Enferm. Infecc. Microbiol. Clin..

[9] Nhinh D.T., Le D.V., Van K.V., Giang N.T.H., Dang L.T., Hoai T.D. (2021). Prevalence, Virulence Gene Distribution and Alarming the Multidrug Resistance of *Aeromonas hydrophila* Associated with Disease Outbreaks in Freshwater Aquaculture. Antibiotics.

[10] Persson S., Al-Shuweli S., Yapici S., Jensen J.N., Olsen K.E.P. (2015). Identification of clinical *Aeromonas* species by rpoB and gyrB sequencing and development of a multiplex PCR method for detection of *Aeromonas hydrophila*, *A. caviae*, *A. veronii*, and, *A. media*. J. Clin. Microbiol..

[11] Vila J., Marcos M.A., Jiménez de Anta M.T. (1996). A comparative study of different PCR-based DNA fingerprinting techniques for typing of the *Acinetobacter calcoaceticus*-*A. baumanii* complex. J. Med. Microbiol..

[12] Chen P.L., Ko W.C., Wu C.J. (2012). Complexity of β-lactamases among clinical *Aeromonas* isolates and its clinical implications. J. Microbiol. Immunol. Infect..

[13] Sunniva Hoel S., Vadstein O., Jakobsen A.N. (2017). Species Distribution and Prevalence of Putative Virulence Factors in Mesophilic *Aeromonas* spp. isolated from Fresh Retail Sushi. Front. Microbiol..

[14] Ottaviani D., Parlani C., Citterio B., Masini L., Leoni F., Canonico C., Sabatini L., Bruscolini F., Pianetti A. (2011). Putative virulence properties of *Aeromonas* strains isolated from food, environmental and clinical sources in Italy: A comparative study. Int. J. Food Microbiol..

[15] Lee H.J., Hoel S., Lunestad B.T., Lerfall J., Jakobsen A. (2020). *Aeromonas* spp. isolated from ready-to-eat seafood on the Norwegian market: Prevalence, putative virulence factors and antimicrobial resistance. J. Appl. Microbiol..

[16] Pablos M., Remacha M.A., Rodríguez-Calleja J.M., Santos J.A., Otero A., García-López M.-L. (2011). Identity, virulence genes, and clonal relatedness of *Aeromonas* isolates from patients with diarrhea and drinking water. Eur. J. Clin. Microbiol. Infect. Dis..

[17] Huddleston J.R., Brokaw J.M., Zak J.C., Jeter R.M. (2013). Natural transformation as a mechanism of horizontal gene transfer among environmental *Aeromonas* species. Syst. Appl. Microbiol..

[18] Nam I.Y., Joh K. (2007). Rapid detection of virulence factors of *Aeromonas* isolated from a trout farm by hexaplex-PCR. J. Microbiol..

[19] Gavin R., Merino S., Altarriba M., Canals R., Shaw J.G., Tomás J.M. (2003). Lateral flagella are required for increased cell adherence, invasion and biofilm formation by *Aeromonas* spp.. FEMS Microbiol. Lett..

[20] Rabaan A.A., Gryllos I., Tomás J.M., Shaw J.G. (2001). Motility and the polar flagellum are required for *Aeromonas* caviae adherence to HEp-2 cells. Infect. Immun..

[21] Igbinosa I.H., Igbinosa E.O., Okoh A.I. (2015). Detection of antibiotic resistance, virulence gene determinants and biofilm formation in *Aeromonas* species isolated from cattle. Env. Sci. Pollut. Res. Int..

[22] Galindo C., Sha J., Fadl A., Pillai L.L., Chopra A.K. (2006). Host Immune Responses to *Aeromonas* Virulence Factors. Curr. Imm Rev..

[23] Albert M.J., Ansaruzzaman M., Talukder K.A., Chopra A.K., Kuhn I., Rahman M., Faruque A.S.G., Islam M.S., Sack R.B., Mollby R. (2000). Prevalence of enterotoxin genes in *Aeromonas* spp. isolated from children with diarrhea, healthy controls, and the environment. J. Clin. Microbiol..

[24] Vila J., Ruiz J., Gallardo F., Vargas M., Soler L., Figueras M.J., Gascon J. (2003). *Aeromonas* spp. and Traveler’s Diarrhea: Clinical Features and Antimicrobial Resistance. Emerg. Infect. Dis..

[25] Ferguson M.R., Xu X.J., Houston C.W., Peterson J.W., Coppenhaver D.H., Popov V.L., Chopra A.K. (1997). Hyperproduction, purification, and mechanism of action of the cytotoxic enterotoxin produced by *Aeromonas hydrophila*. Infect. Immun..

[26] Sha J., Kozlova E.V., Fadl A.A., Olano J.P., Houston C.W., Peterson J.W., Chopra A.K. (2004). Molecular characterization of a glucose-inhibited division gene, gidA, that regulates cytotoxic enterotoxin of *Aeromonas hydrophila*. Infect. Immun..

[27] Sha J., Lu M., Chopra A.K. (2001). Regulation of the cytotoxic enterotoxin gene in *Aeromonas hydrophila*: Characterization of an iron uptake regulator. Infect. Immun..

[28] Asao T., Kozaki S., Kato K., Kinoshita Y., Otsu K., Uemura T., Sakaguchi G. (1986). Purification and characterization of an *Aeromonas hydrophila* hemolysin. J. Clin. Microbiol..

[29] Howard S.P., Buckley J.T. (1982). Membrane glycoprotein receptor and hole-forming properties of a cytolytic protein toxin. Biochemistry.

[30] Wang G., Clark C.G., Liu C., Pucknell C., Munro C.K., Kruk T.M.A.C., Caldeira R., Woodward D.L., Rodgers F.G. (2003). Detection and characterization of the hemolysin genes in *Aeromonas hydrophila* and *Aeromonas sobria* by multiplex PCR. J. Clin. Microbiol..

[31] Pemberton J.M., Kidd S.P., Schmidt R. (1997). Secreted enzymes of *Aeromonas*. FEMS Microbiol. Lett..

[32] Bloch S., Monteil H. (1989). Purification and characterization of *Aeromonas hydrophila* beta-hemolysin. Toxicon.

[33] Palma-Martínez I., Guerrero-Mandujano A., Ruiz-Ruiz M., Hernández-Cortez C., Molina-López J., Bocanegra-García V., Castro-Escarpulli G. (2016). Active Shiga-like toxin produced by some *Aeromonas* spp., isolated in Mexico City. Front. Microbiol..

[34] Alperi A., Figueras M.J. (2010). Human isolates of *Aeromonas* possess Shiga toxin genes (stx1 and stx2) highly similar to the most virulent gene variants of Escherichia coli. Clin. Microbiol. Infect..

[35] Del Valle A., Santos-Pérez J.L., Navarro-Marí J.M., Gutiérrez-Fernández J. (2020). Epidemiological data description of pediatric patients with diarrhea by *Aeromonas* spp. and the antibiotic susceptibility of this agent. Rev. Argent. Microbiol..

[36] Lee J.E., Reed J., Shields M.S., Spiegel K.M., Farrell L.D., Sheridan P.P. (2007). Phylogenetic analysis of Shiga toxin 1 and Shiga toxin 2 genes associated with disease outbreaks. BMC Microbiol..

[37] Leung K.Y., Stevenson R.M. (1988). Tn5-induced protease-deficient strains of *Aeromonas hydrophila* with reduced virulence for fish. Infect. Immun..

[38] Shieh H. (1987). Protection of atlantic salmon against motile aeromonad septicaemia with *Aeromonas hydrophila* protease. Microbios Lett..

[39] Keller T., Seitz R., Dodt J., König H. (2004). A secreted metallo protease from *Aeromonas hydrophila* exhibits prothrombin activator activity. Blood Coagul. Fibrinolysis.

[40] Fernández-Bravo A., López-Fernández L., Figueras M.J. (2019). The Metallochaperone Encoding Gene hypA Is Widely Distributed among Pathogenic *Aeromonas* spp. and Its Expression Is Increased under Acidic pH and within Macrophages. Microorganisms.

[41] O’Halloran T.V., Culotta V.C. (2000). Metallochaperones, an intracellular shuttle service for metal ions. J. Biol. Chem..

[42] Blum F.C., Hu H.Q., Servetas S.L., Benoit S.L., Maier R.J., Maroney M.J., Merrell D.S. (2017). Structure-function analyses of metal-binding sites of HypA reveal residues important for hydrogenase maturation in *Helicobacter pylori*. PLoS ONE.

[43] Huang L., Qin Y., Yan Q., Lin G., Huang L., Huang B., Huang W. (2015). MinD plays an important role in *Aeromonas hydrophila* adherence to *Anguilla japonica* mucus. Gene.

[44] Ji Y., Li J., Qin Z., Li A., Gu Z., Liu X., Lin L., Zhou Y. (2015). Contribution of nuclease to the pathogenesis of *Aeromonas hydrophila*. Virulence.

[45] Suarez G., Khajanchi B.K., Sierra J.C., Erova T.E., Sha J., Chopra A.K. (2012). Actin cross-linking domain of *Aeromonas hydrophila* repeat in toxin A (RtxA) induces host cell rounding and apoptosis. Gene.

[46] Janda J.M., Abbott S.L. (1998). Evolving concepts regarding the genus *Aeromonas*: An expanding panorama of species, disease presentations, and unanswered questions. Clin. Infect. Dis..

[47] Bayerdörffer E., Schwarzkopf-Steinhauser G., Ottenjann R. (1986). New unusual forms of colitis. Report of four cases with known and unknown etiology. Hepatogastroenterology.

[48] Block K., Braver J.M., Farraye F.A. (1994). *Aeromonas* infection and intramural intestinal hemorrhage as a cause of small bowel obstruction. Am. J. Gastroenterol..

[49] Lai C.C., Ding L.W., Hsueh P.R. (2007). Wound infection and septic shock due to *Aeromonas trota* in a patient with liver cirrhosis. Clin. Infect. Dis..

[50] Tena D., Aspiroz C., Figueras M.J., González-Praetorius A., Aldea M.J., Alperí A., Bisquert J. (2009). Surgical site infection due to *Aeromonas* species: Report of nine cases and literature review. Scand. J. Infect. Dis..

[51] Padmaja K., Lakshmi V., Murthy K.V.D. (2013). Sepsis due to *Aeromonas hydrophila*. Int. J. Infect. Control..

[52] Huang T.-Y., Tsai Y.-H., Lee C.-Y., Hsu W.-H., Hsiao C.-T., Huang Y.-K., Li Y.-Y., Chen J.-L., Kuo S.-F., Hsiao J.-C. (2022). Rational Use of Antibiotics and Education Improved *Aeromonas* Necrotizing Fasciitis Outcomes in Taiwan: A 19-Year Experience. Antibiotics.

[53] Aravena-Román M., Inglis T.J., Henderson B., Riley T.V., Chang B.J. (2012). Antimicrobial susceptibilities of *Aeromonas* strains isolated from clinical and environmental sources to 26 antimicrobial agents. Antimicrob. Agents Chemother..

[54] Fosse T., Giraud-Morin C., Madinier I., Labia R. (2003). Sequence analysis and biochemical characterization of chromosomal CAV-1 (*Aeromonas caviae*), the parental cephalosporinase of plasmid-mediated AmpC ‘FOX’ cluster. FEMS Microbiol. Lett..

[55] Zhong Z., Lv X., Gao Y. (2002). *Aeromonas hydrophila* infection. Rev. Med. Microbiol..

[56] Bennett P.M. (2008). Plasmid encoded antibiotic resistance: Acquisition and transfer of antibiotic resistance genes in bacteria. Br. J. Pharmacol..

[57] Stevens D.L., Bisno A.L., Chambers H.F., Everett E.D., Dellinger P., Goldstein E.J.C., Gorbach S.L., Hirschmann J.V., Kaplan E.L., Montoya J.G. (2005). Practice guidelines for the diagnosis and management of skin and soft tissue infections. Clin. Infect. Dis..

[58] Overman T.L., Janda J.M. (1999). Antimicrobial susceptibility patterns of *Aeromonas jandaei*, *A. schubertii*, *A. trota*, and, *A. veronii* biotype veronii. J. Clin. Microbiol..

